# Evolutionary Distances in the Twilight Zone—A Rational Kernel Approach

**DOI:** 10.1371/journal.pone.0015788

**Published:** 2010-12-31

**Authors:** Roland F. Schwarz, William Fletcher, Frank Förster, Benjamin Merget, Matthias Wolf, Jörg Schultz, Florian Markowetz

**Affiliations:** 1 Cancer Research UK Cambridge Research Institute, University of Cambridge, Cambridge, United Kingdom; 2 Department of Genetics, Evolution and Environment and Centre for Mathematics and Physics in the Life Sciences and Experimental Biology, University College London, London, United Kingdom; 3 Department of Bioinformatics, Biocenter, University of Würzburg, Würzburg, Germany; Prognosys Biosciences, United States of America

## Abstract

Phylogenetic tree reconstruction is traditionally based on multiple sequence alignments (MSAs) and heavily depends on the validity of this information bottleneck. With increasing sequence divergence, the quality of MSAs decays quickly. Alignment-free methods, on the other hand, are based on abstract string comparisons and avoid potential alignment problems. However, in general they are not biologically motivated and ignore our knowledge about the evolution of sequences. Thus, it is still a major open question how to define an evolutionary distance metric between divergent sequences that makes use of indel information and known substitution models without the need for a multiple alignment. Here we propose a new evolutionary distance metric to close this gap. It uses finite-state transducers to create a biologically motivated similarity score which models substitutions and indels, and does not depend on a multiple sequence alignment. The sequence similarity score is defined in analogy to pairwise alignments and additionally has the positive semi-definite property. We describe its derivation and show in simulation studies and real-world examples that it is more accurate in reconstructing phylogenies than competing methods. The result is a new and accurate way of determining evolutionary distances in and beyond the twilight zone of sequence alignments that is suitable for large datasets.

## Introduction

State-of-the art phylogenetic reconstruction methods are currently being challenged. For a long time, multiple sequence alignments followed by maximum-likelihood (ML) tree reconstruction have been seen as the computationally expensive gold standard for phylogenetic analyses [1], [2]. Distance approaches that base their inference on summary statistics have traditionally been seen as a fast but less precise alternative [3]. However, recent results point out that the gap between ML and distance methods may be less pronounced than previously thought. For example, the expected required sequence length for the reconstructed tree to converge to the true tree phylogeny is not worse in distance-based approaches than in ML [4]. Additionally the quality of the multiple sequence alignment heavily affects reconstruction accuracy, a situation worsened by the NP-hardness of the alignment problem and the heuristics used to cope with it [5]–[9]. The problem of alignment errors arises especially on large-scale phylogenies with many taxa that span a broad divergence range [10], where many homologies lie in the *twilight-zone* of sequence alignments [11].

In the light of these findings, alignment-free distance-based reconstruction methods deserve special attention, as they circumvent potential pitfalls of the multiple alignment approach, especially with respect to divergent sequences, and can be advantageous in speed possibly without sacrificing reconstruction accuracy. Unfortunately many purely alignment-free approaches [12], [13] lack unique biological motivation (for a comparison see also [14]). Joint estimation of trees and alignments is computationally expensive and relies heavily on heuristics and/or sampling approaches [15]–[19]. The question of reconstructing phylogenies directly without multiple alignment has only recently been tackled [20] with promising results. We follow the basic principles of this approach but here wish to present the phylogenetic reconstruction problem in a different light.

Since there exists a one-to-one relationship between binary trees and additive metrics [21] the phylogenetic problem of finding the true tree is equivalent to finding the true additive dissimilarity matrix. Finding additive distances is hard, thus distance-based approaches usually aim at finding a distance which is as close as possible to the true additive one, so that the tree reconstruction process which turns these non-additive distances into additive trees finds the true tree as often as possible. Metrics in general, including additive distances, can be thought of as being induced by a dot product 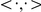 in some Hilbert space of possibly infinite dimension [22]. Key to phylogenetic reconstruction is constructing a Hilbert space and associated dot-product such that distances between sequences are indeed a measure of evolutionary divergence. Doing this explicitly is impossible, if the space is of infinite dimension. However, it can be achieved implicitly by applying the so-called *kernel-trick* [22]: A positive-definite (pd) kernel function 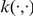 in the input space (i.e. directly on the sequences in our case) computes the scalar value of the dot-product in the Hilbert space without explicitly constructing it.

The kernel trick has been applied successfully in a variety of different fields, including natural language processing, face recognition, speech recognition and computational biology. Here we extend its use to the problem of phylogenetic reconstruction. The major challenge here is finding the right pd kernel. The pairwise similarity measure between sequences must map sequences to an evolutionary feature space ruled by the modification of sequences in terms of insertions, deletions and substitutions. The natural distance in this space should then come as close as possible to the true evolutionary distance on the sequences.

In this article we derive such a kernel. Making use of classical results from global pairwise alignment we show how a different formulation of the alignment problem can map sequences to a feature space of insertions, deletions and substitutions and gives rise to a pd kernel. We study this similarity measure in its topological reconstruction accuracy of phylogenetic trees from simulated and real data. We show its superiority over conventional methods for phylogenetic studies with a broad range of sequence divergence in and beyond the twilight zone of remote homology. We further investigate possible benefits of including suboptimal alignments into the score.

## Materials and Methods

Hidden Markov Models (HMMs) have been extensively used for probabilistic modeling of sequence families, database searches and other tasks. Pair-HMMs work on two sequences simultaneously and are capable of probabilistic modeling of pairwise alignments [23]. The field of natural language processing uses close relatives of HMMs, so called *finite-state transducers (FST)*, for modeling the transformation of one sequence into another or describing joint distributions on two sequences [24]. Their advantage over pair-HMMs is the rigorous and general definition which allows not only for probabilistic interpretations, but for any set of values that follows specific rules (more precisely all *semirings*) to be used as weights. These include, for example: probabilities, logarithmic numbers (where the weights are summed along a path instead of multiplied); and boolean values. In the following we use FSTs to create our kernel for evolutionary sequence comparison. We make use of two major observations: (i) The classical problem of pairwise alignment can be posed as a shortest-path problem on a log-weighted FST [25]; and (ii) FSTs that can be decomposed into another FST and its inverse give rise to pd rational kernels [26].

### Semirings

The different classes of weights that can be used for FSTs are the so-called semirings. They define two operations on a set, an abstract sum and multiplication. In the case of the *real* semiring, the final score for two sequences is the (conventional) sum of all possible paths generating those two sequences, where the weights in each path are (conventionally) multiplied. Weights on the *real* semiring can be converted to the *log* semiring by the link function 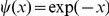. In the *log* semiring, multiplication is turned into summation and the sum is replaced by the logarithm of the sum. The *tropical* semiring is a special instance of the *log* semiring in that the log-sum over all paths is replaced by the *minimum*, and corresponds to the *Viterbi* approximation in conventional HMMs. For a more formal definition of semirings, see Text S1 or [26], [27] and references therein.

### Alignment Problems as FSTs

Any edit-distance can be computed via a FST over the *tropical* semiring [26]. This includes the classical edit-distance [28] as well as any generalized alignment problem. The alignment score is then the minimum of all possible paths of transforming one sequence into another. More formally, for a FST 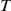 over the *tropical* semiring, the alignment score is defined as 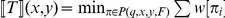, where 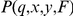 is the set of all paths going from the initial states 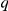 to the final states 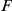 thereby transforming 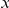 to 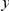. The standard global pairwise alignment problem for example is a three state FST with a map, an insert and a delete state. The self-transitions in the match state are weighted with the scores of the used substitution matrix, the transitions to the gap states and the self-transitions in the gap state are weighted with the gap open/gap extend costs respectively.

### PD Rational Kernels and distances

A FST 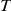 over the *real* semiring associates a real-valued number with every pair of sequences 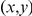. This score is then the *sum* over all possible paths transforming 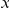 to 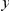, *multiplying* instead of summing the weights along the path. This mapping from two-tuples of the space of sequences to the reals is called a rational kernel. If the transducer 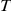 can further be decomposed into a transducer 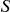 and its inverse 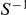 (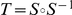), the kernel is known to be pd [26] (for details on FST composition and inversion see Text S1 or [27]). In this setting, the transducer 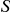 performs the feature space mapping. It encodes the prior knowledge about the features important for our problem domain. From a pd kernel we can directly compute distances in the feature space via 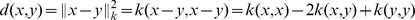.

### Pairwise alignments as pd rational kernels

It is our goal to modify the pairwise alignment problem in a way that we can prove the resulting alignment score to be pd. To achieve this we replace the min operation by the log-sum, thereby changing semirings from the tropical to the log. The resulting score includes all possible (suboptimal) alignments. By making use of the link function 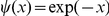 we can convert that logarithmic score into a real value. The result is the score of a rational kernel [26].

**Figure 1:**
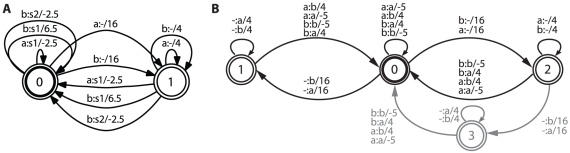
Feature space mapping and kernel function transducers for the evolutionary sequence space. *A:* Feature space mapping for biological sequences using a FST over the log semiring: Every transition has an attached input and output symbol separated by a colon, and an associated weight. Symbols can either be kept, substituted or deleted. Composition of such a transducer with its own inverse yields a pd rational kernel. The alphabet has been reduced to two symbols for illustration purposes, 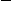 depicts a gap or epsilon transition. *B:* Result of the composition of the transducer encoding the feature space mapping with its inverse: The starting state (state 0) corresponds to the match state, the additional two colored states (states 1, 2) encode insertion and deletion states. The transitions to the gap states are scored with gap open costs and the self transitions in the gap state with gap extend costs. The additional fourth state (3) is a result of the epsilon filtering process during composition.

To see that this kernel is indeed pd we need to decompose it in to a feature space mapping FST and its inverse 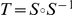. On the real semiring and ignoring epsilon transitions (gaps), it is easy to see that by the definition of composition this equals a Cholesky Decomposition of the transition weight matrix, which requires the pointwise exponential of the substitution matrix used to be pd. If we wish to include gaps we need to construct the feature space explicitly:

We can think of a feature space mapping where each position in a biological sequence can either be retained, substituted or deleted using some intermediate alphabet 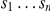. For an example of such a FST with weights derived from a standard nucleotide substitution matrix and gap scores of 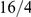 see Figure 1a. Composition of this FST with its own inverse, obtained by reversing input and output symbols, leads to the FST in Figure 1b.

It can easily be seen how the composed FST again resembles the topology of a global alignment FSA [23], with a match state and two states corresponding to insertions or deletions. The additional fourth state contained in the transducer is a result of the epsilon filter used. Different epsilon filters lead to different topologies [24], [27] where the three-state backbone of match, insertion and deletion states are always retained. This additional path theoretically allows for the opening of a new gap within a gap, something which is automatically excluded if looking for the shortest path or best-scoring alignment between two sequences.

In summary, reformulation of the classical global pairwise alignment paradigm allows for the interpretation of the alignment score as a shortest-path approximation of the kernel score of a pd rational kernel working on biological sequences.

### The impact of suboptimal alignments on the kernel score

If the absolute difference between the summands of a logarithmic sum is large the sum is heavily dominated by its smaller summand. Therefore, in cases where the optimal alignment score is distinctively smaller than any suboptimal alignment the kernel score including all suboptimal alignments will be close to the shortest-path approximation. In cases where even the best alignment score is not significantly smaller than its closest suboptimal siblings the full score will differ. In order to be able to study the effect of the inclusion of suboptimal alignments in terms of reconstruction accuracy we project the exponential of the matrix of pairwise alignment scores to the next positive semi-definite [29]. This shortest-path approximation is not neccesarily pd anymore. How big the difference is depends on the optimality of the best score as discussed above.

## Results

We performed repeated simulation experiments to validate our distance measures using nucleotide and amino acid sequences over two different tree topologies and on each with increasing sequence divergence.

### Sequence simulation

Amino acid and nucleotide sequences were generated according to two tree topologies with 18 and 52 taxa in realistic scenarios using INDELible [30] (see also Text S1). Trees were reconstructed and topologically compared to the true tree using the quartet distance [31]. The studied methods were (i) traditional multiple alignment using *Muscle* [32] followed by Jukes-Cantor distance estimation using *Phylip* [33], (ii) statistical consistency alignment using *ProbCons* based on pair-HMMs [34] followed by *RAxML* maximum-likelihood tree reconstruction [35], (iii) an alignment-free method of distance estimation based on the Lempel-Ziv complexity [12], (iv) a pattern-based maximum-likelihood approach for alignment-free distance estimation [36] and (v) the classical Levenshtein distance [28]. Comparison according to (iv) had to be performed on a much smaller sample size due to the high computational demand of the method [14]. In a preliminary study we found (iv) to perform only slightly better than (iii) for closely related sequences. We thus kept method (iii) as a representative for alignment-free methods.

### Sequence divergence leads to poor alignment quality

**Figure 2:**
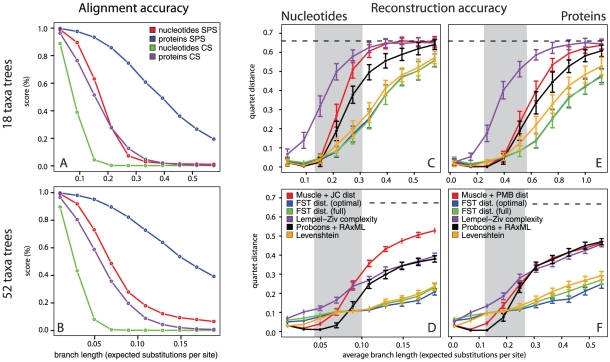
Alignment accuracy results and reconstruction accuracy results on simulated sequences with increasing divergence. *A,B:* Alignment accuracies measured in column and sum-of-pairs scores. With increasing branch lengths multiple alignments accumulate errors, which leads to the poor reconstruction accuracies observed. *C–F:* Simulation results for nucleotide sequences (left) and protein sequences (right): All experiments were repeated 100 times, standard error estimates are shown. The traditional approach of multiple alignment followed by distance estimation performs well for closely related sequences (red and black lines). The error curve of the two FST approaches has a significantly lower slope and performs well even for divergent sequences (green and blue), so does the classical edit-distance (yellow) which is still behind the FST distances. Statistical consistency aligners (black) perform better than traditional aligners (red) but suffer from the same rapid decay in reconstruction accuracy. The Lempel-Ziv complexity-based distance only achieves good results for the 52 taxa tree (purple). The dotted black line at the top gives the maximum expected quartet distance from a random tree.

To assess the impact of sequence divergence on multiple alignment accuracy we first compared the alignments from Muscle with the true INDELible alignments. We calculated two scores to quantify this accuracy (Figure 2 A,B): The *column score* (CS) is the proportion of columns from the true alignment that are present and correct in the test alignment. The *sum-of-pairs score* (SPS) is the proportion of aligned pairs of nucleotides/amino-acids from the true alignment that are also aligned together in the test alignment. The first is a very stringent measure as all nucleotides/amino-acids in a column must be correctly placed for that column to be deemed correct. The latter is a more lenient measure as it rewards correct alignment between some sequences even if other sequences in that column are mis-aligned [9].

The results show that the number of correctly aligned positions exponentially decreases with increasing sequence divergence for nucleotide sequences. Amino acid sequences showed a more linear trend, possibly due to the higher information content introduced by the larger alphabet size of amino acids as compared to nucleotides, but suffer from the same effect.

### FST distance for divergent sequences

Quartet distances between the estimated and true trees for nucleotide as well as amino acid sequences over all tree topologies (Figure 2, C–F) show that the traditional approach of a multiple alignment followed by distance estimation is highly accurate for closely related species. When entering the twilight zone of sequence alignments reconstruction accuracy drops exponentially. Above average branch lengths of 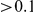 substitutions per site for the 52 taxa nucleotide tree and 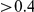 substitutions per site for the 18 taxa protein tree the multiple alignment becomes erroneous (red and black lines) and tree reconstruction accuracy gets weak as the number of quartets in common with the true tree approaches that of a random tree (dotted line). This effect is about 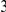 times stronger for nucleotide then for protein trees and about 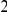 times stronger in the 52-taxa tree as compared to the 18-taxa tree. To exclude aligner-specific artifacts, we included ProbCons (black line, Figure 2) into the analysis. We additionally included ClustalW which was found to perform slightly worse than Muscle. We also computed maximum-likelihood trees on the alignments to assess the difference in reconstruction accuracy between simple distance-based and character-based approaches. The RAxML trees outperformed the distance based trees by a margin which was more profound for amino acid sequences than for nucleotide sequences, but still suffered drastic loss in reconstruction accuracy with increasing sequence divergence, due to the accumulation of alignment errors. The *ProbCons* results showed that aligners specifically designed to address these issues indeed perform slightly better to moderately better across all experiments but still suffer from a rapid loss in reconstruction accuracy with increasing sequence divergence. The alignment-free methods generally performed worse than other methods tested on the 18 taxa tree but were close to the best alignment-based methods for the 52 taxa trees.

Distance estimation using our proposed finite-state transducers (blue and green lines) came close to the performance of classical multiple alignment for closely related species. It showed only a gradual decrease in reconstruction accuracy with increasing evolutionary divergence, being significantly more accurate than any other method tested. This evidently shows that the classical approach of multiple alignment followed by tree reconstruction is superior only if the alignment is correct. From a certain distance on, multiple sequence alignments cannot be reconstructed accurately any more, leading to poor reconstruction power in the downstream phylogenetic analysis. This seems to hold for classical progressive multiple alignment as well as statistical consistency alignment.

### The influence of suboptimal alignments on the kernel score

When comparing both proposed kernel scores, one incorporating all suboptimal alignments into the score, the other only using the optimal alignment, we noticed differences between the two tree topologies: In the 18-taxa case both variants perform equally well. In the 52-taxa case the suboptimal alignments added more noise to the score than signal and the kernel using only the optimal score came out ahead. Even though the average branch length in the 52 taxa tree is moderate the variance of pairwise distances between sequences is higher when the tree contains more branches. When comparing sequences beyond the twilight zone the scores of the optimal and subsequent suboptimal alignments are similar, influencing the kernel score (see *Methods* section). The good performance of the score based on the optimal alignment is especially attractive as this shortest-path approximation can be computed with standard global alignment implementations, such as the Stretcher program from the EMBOSS package followed by projection to the next pd matrix. We were already able to test this procedure in a real-world application, comparing 500 human kinases with 2600 kinases from *Paramecium tetraurelia*. We showed that the kinome of *P. tetraurelia* is more than 5 times the size of the human kinome. In addition to whole genome duplications, further duplications lead to the expansion of specific subfamilies. More than 20 ciliate specific domain architectures were discovered [37].

The most traditional way of pairwise comparison between sequences is the edit-distance or Levenshtein distance [28]. Results show, that like our own pairwise sequence comparisons the Levenshtein distance is not prone to the multiple alignment pitfall and therefore performs well for divergent sequences. It provides a relatively accurate estimator for distances between nucleotide sequences but performs worse for protein sequences. This is not surprising as the EDNAFULL matrix traditionally used for nucleotide alignments scores matches with 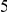 and all substitutions equally with 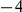. The information content of this matrix is not higher than that of the edit-distance matrix which scores matches with 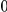 and any edit operation with 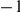. The picture changes in the case of protein substitution matrices that carry dense information about the exchangeability of amino acids.

### FST distance places *Sphaeroplea* clade correctly without information about secondary structures

We applied our method to a set of 52 internal transcribed spacer II (ITS2) sequences of the Chlorophyceae [38]. The group consists of 6 major clades of high within-group sequence similarity (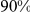 median pairwise sequence identity) but significant divergence between groups (down to 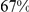 total average sequence identity). Over the last few decades there has been ongoing discussion about placement of the *Sphaeroplea* clade within this set of taxa [39]–[41]. Even though most authors agree on the existence of a monophyletic *DO*-group comprising the *Sphaeroplea*, *Hydrodictyon* and *Scenedesmus* clades, the position of the *Sphaeroplea* clade within this group was only recently verified by taking structural properties of the ITS2 into account [38].

**Figure 3:**
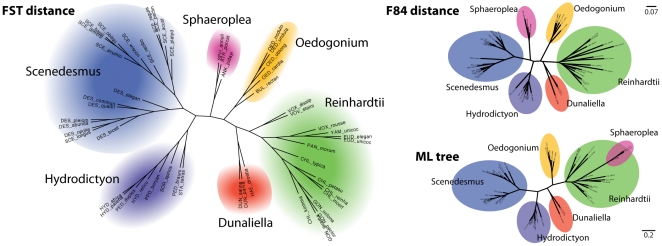
Reconstructed phylogenetic trees of the Chlorophyceae group. Three different methods were compared: FST distance (left) using the full kernel score, F84 distance estimation on a Muscle alignment (top right) and maximum-likelihood tree on the same Muscle alignment (bottom right). Only the FST tree reveals the same grouping of the major clades as discussed in [38], which we use as a ‘gold standard’. The distance tree erroneously places the *Sphaeroplea* clade between the *Hydrodictyon* and *Scenedesmus* clades, while the ML tree places them within the *reinhardtii*-subgroup.

We applied our FST distance method to the set of ITS2 sequences and compared it to both classical distance estimation and maximum likelihood tree reconstruction on a multiple sequence alignment (Figure 3). Comparing the MSA with the manually curated sequence structure alignment taken from [38] shows that the Muscle alignment contains many misaligned columns (CS 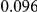, SPS 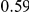). The reconstructed trees differ from the true tree, especially with respect to the placement of the *Sphaeroplea* clade. The distance tree places the *Sphaeroplea* clade between the *Hydrodictyon* and *Scenedesmus* clades (Figure 3 right top), whereas the ML tree again places the *Sphaeroplea* clade within the *reinhardtii*-subgroup (Figure 3 right bottom). Our FST distance, which circumvents the multiple alignment step, correctly places the *Sphaeroplea* clade next to the *Hydrodicton/Scenedesmus* sister clade. In other methods this position can only be inferred by using additional secondary structure information to reduce alignment errors. Our method was additionally able of correctly grouping a monophyletic *Gonium* clade.

## Discussion

In this paper we have shown that a kernel-based distance measure circumvents problems of MSA quality and performs very well in and beyond the twilight zone of remote homology. We intentionally used known substitution matrices and gap scores as parameters to illustrate the link to classical global alignments. Custom parameters estimated by e.g. expectation maximization over alignments of a given divergence range will supposedly perform even better.

Using FSTs to derive the distance has several advantages. For example, the inputs to the distance calculation are currently two individual sequences, formulated as finite-state acceptors that emit exactly the sequence under study. This can seamlessly be extended to acceptors emitting distributions over sequences, i.e. Hidden Markov Models like profile-HMMs [42] to compute distances between sequence families with possible applications in e.g. Profile-Neighbor-Joining [43]. The construction of a pd kernel using composition of two individual FSTs is a necessary step, as generalized edit-distances like the classical pairwise alignment score, are not negative definite and therefore can not easily be turned into a pd kernel by exponentiation alone [26].

The methods we compare ourselves against are amongst the most frequently applied, such as *Muscle* followed by a distance-based tree reconstruction, but also include state-of-the art statistical consistency aligners like ProbCons followed by ML tree reconstruction. As both JC distance and ML reconstruction methods suffer from the same decrease in accuracy we show that it is not simply the modeling of insertions and deletions that is improved in our distance measure as compared to JC. We additionally clustered sequences by length to see if the sheer number of insertions and deletions in divergent sequences were mainly responsible for this effect. This clustering performed very poorly as expected.

Sophisticated methods of statistical alignment [44] are capable of computing joint probabilities for sequence comparison, but the derivation of distances remains arbitrary to a degree. Furthermore, such statistical methods can be found lacking because of simplifications such as assuming that indel events involve only one residue (TKF91 model, [15]) or that sequences are made from non-overlapping indivisible fragments (TKF92 model, [16]). Maximum-likelihood estimates for the time elapsed between two species given the sequences additionally involve reversibility assumptions and solving non-convex optimization problems.

Other algorithms, such as [45], are only practical in analyses involving a small number of sequences. They necessarily need to be coupled to numerical optimization methods to find maximum likelihood estimates of parameters such as insertion and deletion rates, substitution parameters, and branch lengths. In contrast, our approach is capable of directly using substitution matrices that are known to perform well for certain evolutionary distances.

In summary, the present fast and MSA-free methodology allows us to compute pairwise distances between sequences that mirrors the global pairwise alignment process. Our methodology interprets alignment scores as values of a kernel that implicitly maps sequences to a feature space with a biologically motivated topology: it is built of modifications of that sequence using insertions, deletions and substitutions. Our methodology can directly be applied to compute distances between distributions of sequences. The resulting pd kernel matrix can be used in any method that can be expressed in terms of dot products alone (e.g. classification via support vector machines). The distances are meaningful in evolutionary terms and outperform other phylogenetic inference methods on divergent sequences in and beyond the so-called twilight zone of remote homologies. Thus, our methods complement traditional approaches for more closely related sequences. Future work will focus on assessing the robustness of the kernel score (bootstrapping) and the question of mapping sequences directly to an additive space, i.e. from which additive distances can be immediately derived, to remove the final approximation step when going from the matrix of pairwise distances to the tree.

## Supporting Information

Text S1 Supporting text.
(PDF)
