# Supplementary material for: Evolutionary Distances in the Twilight Zone—A Rational Kernel Approach
Source: PLoS One. 2010 Dec 31;5(12):e15788. doi: 10.1371/journal.pone.0015788 (PMC3013127; doi:10.1371/journal.pone.0015788)
Supplement: Text S1 — Supporting text. (PDF) [file pone.0015788.s001.pdf]

Evolutionary distances in the twilight zone – a rational kernel approach
SUPPORTING INFORMATION

Roland F. Schwarz[1,*], William Fletcher[2], Frank Förster[3], Benjamin Merget[3], Matthias Wolf[3], Jörg Schultz[3], Florian Markowetz[1,*]
1 CRUK Cambridge Research Institute, University of Cambridge, Cambridge, UK
2 Department of Genetics, Evolution and Environment and Centre for Mathematics and Physics in the Life Sciences and Experimental Biology, University College London, London, UK
3 Department of Bioinformatics, Biocenter, University of Würzburg, Würzburg, Germany
∗ E-mail: rfs32@cam.ac.uk, florian.markowetz@cancer.org.uk

Text S1

Sequence generation In all experiments, sequences were generated with *INDELible* [1] over 10 increasing steps of sequence divergence. Two representative tree topologies were considered: (i) an artificial tree of 18 species with ± 50% varying branch lengths [2] and (ii) a genuine tree of 52 species of the order Sphaeropleales [3]. The branch lengths of the trees were normalized such that the total sum of all branches of the tree equals 1 for the first simulation step. This tree length was then increased by 2 for every simulation step above the first up to a maximum of 19. For the 18 taxa tree this corresponds to an average branch length ranging from 0.03 for the initial tree to 0.63 for the most divergent tree. For the 52 taxa tree the average branch lengths range from 0.01 to 0.19. The indel model used generated indels at a rate of 1 indel per 10 substitutions with insertions and deletions equally likely to occur. Indel lengths were modeled with a geometric distribution where an indel of length u occurs with a probability of $(1 - q)q^{(u-1)}$ with $q = 0.35$. This indel rate is similar to published estimates (e.g. [4]) and the length distribution provides an adequate/reasonable fit to published data for indels in protein coding sequences in mammals (e.g. [5]) and has been used in other simulation studies [6]. All experiments were performed using nucleotide as well as protein sequences, using Jukes-Cantor [7] and Blosum substitution models [8] for the generation of sequences. The sequences had a length of 600 nucleotides or 200 amino acids and each experiment was repeated 100 times.

Method comparison The generated sequences were passed on to the different methods used to reconstruct phylogenetic trees. Multiple alignments were performed with *Muscle 3.7* [9] and *ProbCons 1.12* [10], distance matrices were successively computed with *DNADIST* from the Phylip 3.68 package [11]. We used Jukes-Cantor and PMB (Probability Matrix from Blocks, [12]) distance measures for reconstructing trees to be as similar as possible to the sequence generating process. Fast maximum-likelihood tree reconstruction was performed using *RAxML 7.0.4* [13], using the GTRGAMMA and PROTGAMMABLOSUM62 substitution models for nucleotide and protein trees respectively. Other related substitution models as well as estimation of base frequencies from the alignment in RAxML were tested but did not make a significant difference in terms of topological reconstruction accuracy (data not shown). Pairwise global and semi-global alignments were computed using *Needle* and *Stretcher* from the EMBOSS 6.0.1 package [14]. The scoring matrices used were EDNAFULL for DNA, and Blosum62 for protein sequences as contained in the EMBOSS package. We used affine gap cost models with gap open costs of 10 and gap extend costs of 1. In general, the same or the most similar available scoring matrices and gap costs were used for all external programs and our own transducer-based approach to make results as comparable as possible. All trees from distance matrices were reconstructed using BioNJ [15]. To determine accuracy of the tree reconstruction method, quartet distances between the reconstructed and the original trees were computed using *QDist* 2.0.2 [16]. Computations were carried out in parallel on a

160 core computer cluster (80 Xeon 5140 CPUs, 448 GB RAM in total) running SUSE Linux Enterprise Server 10 (x86-64) as operating system. The total calculation time was about 7350 CPU hours.

Semirings A system $(\mathbb{K}, \oplus, \otimes, \overline{0}, \overline{1})$ is called a semiring if $(\mathbb{K}, \oplus, \overline{0})$ and $(\mathbb{K}, \otimes, \overline{1})$ form monoids, i.e. they are closed under $\oplus$ and $\otimes$ and have $\overline{0}$ and $\overline{1}$ as their identity elements respectively. Both are associative, $(\mathbb{K}, \oplus, \overline{0})$ is further commutative. The most prominent semirings are the *log* semiring $(\mathbb{R} \cup \{-\infty, +\infty\}, \oplus_{\log}, +, +\infty, 0)$, where $p_1 \oplus_{\log} p_2 = -\log(e^{-p_1} + e^{-p_2})$ is the sum in the log space and which is isomorphic to $\mathbb{R}$ via the semiring morphism $y = e^{-x}$, and the *tropical* semiring $(\mathbb{R} \cup \{-\infty, +\infty\}, \min, +, +\infty, 0)$.

Finite-state transducers A weighted finite-state transducer over a semiring[1] $\mathbb{K}$ is a 8-tuple $T = (\Sigma, \Delta, Q, I, F, E, \lambda, \rho)$ where Σ is the finite input alphabet and Δ is the finite output alphabet. Q is a finite set of states of which $I \subseteq Q$ is the set of initial states and $F \subseteq Q$ is the set of final states. $E \subseteq Q \times (\Sigma \cup \epsilon) \times (\Delta \cup \epsilon) \times \mathbb{K} \times Q$ is a finite set of transitions, $\lambda : I \to \mathbb{K}$ the initial weight function and $\rho : F \to \mathbb{K}$ the final weight function. Like other finite-state machines, finite-state transducers can be depicted graphically (for an example, see main text).

Using the $\oplus$-sum and $\otimes$-product of the semiring $\mathbb{K}$, a transducer assigns an output weight to any pair of input-output strings (x, y) via

$$[\![T]\!](x, y) = \bigoplus_{\pi \in P(I,x,y,F)} \lambda(p[\pi]) \otimes w[\pi] \otimes \rho(n[\pi]),$$

i.e. the $\oplus$-sum over all possible paths transforming string x into string y, thereby going from the initial to the final states.

When the semiring $\mathbb{K}$ is the *tropical* semiring the final output weight of the transducer is the minimum over all possible paths transforming x to y (the Viterbi approximation).

For any transducer T, its inverse T^{-1} is defined by the transducer obtained by exchanging input and output symbol at every transition and exchanging the input and output alphabets.

Two transducers $T_1 = (\Sigma^*, \Delta^*, Q_1, I_1, F_1, E_1, \lambda_1, \rho_1)$ and $T_2 = (\Delta^*, \Omega^*, Q_2, I_2, F_2, E_2, \lambda_2, \rho_2)$ can be composed to form a new, usually more complex, transducer, where the composition is defined as:

$$[\![T_1 \circ T_2]\!] = \bigoplus_{z \in \Delta^*} [\![T_1]\!](x, z) \otimes [\![T_2]\!](z, y).$$

The resulting transducer is of the form $T = (\Sigma^*, \Omega^*, Q, I, F, E, \lambda, \rho)$, where the states of T can be identified with pairs of a state of T_1 and a state of T_2, $Q \subseteq Q_1 \times Q_2$. In the same sense, initial states are states which are initial in both T_1 and T_2. Final states are defined equivalently. Transitions are obtained by matching transitions of T_1 with transitions of T_2 in which output symbols of the T_1 transitions have to be input symbols of the T_2 transitions. So, for two transitions $e_1 = (q_1, a, b, w_1, q_2) \in E_1$ and $e_2 = (q_1', b, c, w_2, q_2') \in E_2$, the composed transition is $e = ((q_1, q_1'), a, c, w_1 \otimes w_2, (q_2, q_2'))$. Note that the $\otimes$-product depends on the semiring used.

Rational kernels A function K over two non-empty sets X and Y defining the map $K : X \times Y \to \mathbb{R}$ is called a *kernel*. A kernel K over $\Sigma^* \times \Delta^*$ is a *rational kernel*, if there exists a weighted transducer $T = (\Sigma, \Delta, Q, I, F, E, \lambda, \rho)$ over the semiring $\mathbb{K}$ and a function $\psi : \mathbb{K} \to \mathbb{R}$ such that for all $x \in \Sigma^*$ and $y \in \Delta^*$

$$K(x, y) = \psi([\![T]\!](x, y))$$

defines the kernel function. A rational kernel K is further symmetric positive semi-definite (PSD), iff it can be decomposed into another transducer S and its inverse S^{-1} via $T = S \circ S^{-1}$ [18].

[1]The implementations used apply more precisely to a subset, the so-called *locally closed semirings*, for details see [17].

The problem of scoring two sequences with a transducer (or a rational kernel) is equivalent to solving the *single-source shortest distance problem* of the transducer composed with its input and output strings in form of finite-state acceptors [19].

In this paper we used the natural link function $\psi = exp(-t[\![T]\!](x,y))$ for some normalizing constant $0 < t < 1$.

Implementation All scripting, parsing of file formats and data handling implemented in Python 2.6 using BioPython 1.54 [20]. All statistical analyses, projection to next positive semi-definite and plotting done in R 2.11.0 [21]. All finite-state transducers implemented using the OpenFST library [22].

References

1. Fletcher W, Yang Z (2009) INDELible: a flexible simulator of biological sequence evolution. Mol Biol Evol 26: 1879–1888.

2. Keller A, Förster F, Müller T, Dandekar T, Schultz J, et al. (2010) Inlcuding RNA Secondary Structures improves Accuracy and Robustness in Reconstruction of Phylogenetic Trees. Biology Direct 5: 4.

3. Keller A, Schleicher T, Förster F, Ruderisch B, Dandekar T, et al. (2008) ITS2 data corroborate a monophyletic chlorophycean DO-group (Sphaeropleales). BMC Evol Biol 8: 218.

4. Ogurtsov AY, Sunyaev S, Kondrashov AS (2004) Indel-based evolutionary distance and mouse-human divergence. Genome Res 14: 1610–1616.

5. Taylor MS, Ponting CP, Copley RR (2004) Occurrence and consequences of coding sequence insertions and deletions in Mammalian genomes. Genome Res 14: 555–566.

6. Fletcher W, Yang Z (2010) The Effect of Insertions, Deletions and Alignment Errors on the Branch-Site Test of Positive Selection. Mol Biol Evol 27(10): 2257-2267.

7. Jukes T, Cantor C (1969) Mammalian Protein Metabolism. Academic Press, New York, 21-132 pp.

8. Henikoff S, Henikoff JG (1992) Amino acid substitution matrices from protein blocks. Proc Natl Acad Sci U S A 89: 10915–10919.

9. Edgar RC (2004) MUSCLE: multiple sequence alignment with high accuracy and high throughput. Nucleic Acids Res 32: 1792–1797.

10. Do CB, Mahabhashyam MSP, Brudno M, Batzoglou S (2005) ProbCons: Probabilistic consistency-based multiple sequence alignment. Genome Res 15: 330–340.

11. Felsenstein J (2009). PHYLIP (Phylogeny Inference Package) version 3.6. Distributed by the author. Department of Genome Sciences, University of Washington, Seattle.

12. Veerassamy S, Smith A, Tillier ERM (2003) A transition probability model for amino acid substitutions from blocks. J Comput Biol 10: 997–1010.

13. Stamatakis A (2006) RAxML-VI-HPC: maximum likelihood-based phylogenetic analyses with thousands of taxa and mixed models. Bioinformatics 22: 2688–2690.

14. Rice P, Longden I, Bleasby A (2000) EMBOSS: the European Molecular Biology Open Software Suite. Trends Genet 16: 276–277.

15. Gascuel O (1997) BIONJ: an improved version of the NJ algorithm based on a simple model of sequence data. Mol Biol Evol 14: 685–695.

16. Mailund T, Pedersen CNS (2004) QDist–quartet distance between evolutionary trees. Bioinformatics 20: 1636–1637.

17. Esik Z, Kuich W (2002) Locally Closed Semirings. Monatsh Math 137: 21-29.

18. Cortes C, Haffner P, Mohri M (2004) Rational Kernels: Theory and Algorithms. JMLR 1: 1-50.

19. Droste M, Kuich W, Vogler H, editors (2009) Handbook of Weighted Automata, Springer, chapter Weighted Automata Algorithms. pp. 1-45.

20. Cock PJA, Antao T, Chang JT, Chapman BA, Cox CJ, et al. (2009) Biopython: freely available Python tools for computational molecular biology and bioinformatics. Bioinformatics 25: 1422–1423.

21. "R Development Core Team" (2008) R: A Language and Environment for Statistical Computing. R Foundation for Statistical Computing, Vienna, Austria. URL `http://www.R-project.org`. ISBN 3-900051-07-0.

22. Allauzen C, Riley M, Schalkwyk J, Skut W, Mohri M (2007) OpenFst: A General and Efficient Weighted Finite-State Transducer Library. Proceedings of the Ninth International Conference on Implementation and Application of Automata, (CIAA), in Lecture Notes in Computer Science 4783: 11-23.
